# Heart Disease in Children

**Published:** 1902-02-08

**Authors:** 


### HEART DISEASE IN CHILDREN.

In his presidential address, delivered before the Harveian Society, Dr. D. B. Lees took for his text, "The Heart of the Child," and he enforced the importance of the subject by the striking statement that if a child is to survive for 50 years its heart has before it an enormous task, equal to the lifting of 1,500,000 tons to a height of 1 foot. On its continued efficiency and its capacity for doing each day its allotted share of this great work the man's efficiency largely, perhaps mainly, depends. Among the many topics alluded to by Dr. Lees we will confine ourselves to what he says about acute dilatation of the right auricle. What we are able to find out about a heart by percussion is the size of the left ventricle and of the right auricle. Very few percussors, says Dr. Lees, pay the least attention to the latter structure, and many seem to be unaware of the fact that the dulness due to it may always be detected, even in the normal heart, in the fourth intercostal space. The third space ought to be resonant quite up to the sternum, and in the fifth space the hepatic dulness alters the note, but in the fourth space the dulness of the right auricle is present for about one finger-breadth in an adult and rather less in a child. But when the auricle is dilated this dulness may extend to two finger-breadths in the fourth space, and from a half to one finger-breadth in the third. When the dilatation is very great the dulness may be even more extensive. The accurate determination of the size of the right auricle is a matter of the greatest importance, and often indicates at once the necessity for leeches to relieve distension. Yet there is no part of physical examination which is so universally and systematically neglected, the patient being too often allowed to suffer cardiac distress when he might be relieved in a few minutes. "The suffering due to a distended bladder a medical man would promptly relieve, but that due to a distended right heart is allowed to remain because his percussion is not accurate and bleeding is out of fashion." In auscultating the heart of a child one must be on the look-out for some very puzzling murmurs due to congenital malformation. They are mostly systolic in time, a presystolic or diastolic congenital murmur being exceedingly rare.

The most important affection of the right heart clinically is secondary to acute, or extensive chronic, disease of the lungs, or to disease of the left heart. In all such conditions a careful watch should be kept on the degree of distension of the right auricle. If this distention exceed a certain amount the border line which separates safety from danger is easily crossed. Distress and dyspnœa are experienced and the right ventricle has greater and greater difficulty in expelling its blood. If at the same time its muscular substance is poisoned by pneumococcal toxins it is easy to see that even in a child, whose other organs may be healthy, a pneumonia is a process dangerous to life. Doubtless the child has a much better chance than the adult, yet even in a child the violence of the storm may be much diminished and the patient may be often very obviously relieved by applying a few leeches over his liver. Not only are the distress and dyspnœa diminished, but the patient is enabled to obtain sleep, which with a distended right heart may have been impossible. Within a short time after the bleeding one may find, on careful percussion, that the dulness in the fourth right space has diminished, often by as much as a finger-breadth, and this relief will almost certainly last for two days, perhaps for longer. Each day, however, the auricular dulness must be carefully percussed out, for if the pneumonia continue to increase it may be necessary to repeat the leeches in two or three days, and in some cases this may be necessary even a third time. Pallor of face and smallness of pulse are not necessarily contraindications, for when the right heart has been relieved the pulse will be stronger and the colour of the cheeks will improve. This matter of attention to the condition of the right auricle is of importance not only in pneumonia but in many other affections. In acute bronchitis, in chronic bronchitis with an acute exacerbation, in whooping cough with its mixture of pulmonary collapse, broncho-pneumonia and emphysema, and in asthmatic attacks, the condition of the right auricle may give the key to treatment, as it may also in all cases of disease of the left side of the heart, especially when it has been injured by rheumatism.

